# Spatial Distribution and Environmental Significance of Phosphorus Fractions in River Sediments and Its Influencing Factor from Hongze and Tiaoxi Watersheds, Eastern China

**DOI:** 10.3390/ijerph17165787

**Published:** 2020-08-10

**Authors:** Ja Bawk Marip, Xuyin Yuan, Hai Zhu, Isaac Kwesi Nooni, Solomon O. Y. Amankwah, Nana Agyemang Prempeh, Eyram Norgbey, Taitiya Kenneth Yuguda, Zaw Myo Khaing

**Affiliations:** 1 Key Laboratory of Integrated Regulation and Resources Development of Shallow Lakes of Ministry of Education, College of Environment, Hohai University, Nanjing 210098, China; zhuhai1310@163.com (H.Z.); eyramnorgbey@outlook.com (E.N.); taitiyayuguda@outlook.com (T.K.Y.); zmkhine2011@gmail.com (Z.M.K.); 2 Fujian Provincial Key Laboratory of Eco-Industrial Green Technology, College of Ecology and Resources Engineering, Wuyi University, Wuyishan 354300, China; 3 Binjiang College of Nanjing University of Information Science and Technology, No. 333, Xishan Road, Wuxi 214105, China; nooni25593@alumni.itc.nl; 4 School of Geographical Sciences, Nanjing University of Information Science and Technology, Nanjing 210044, China; 20195111003@nuist.edu.cn; 5 School of Geosciences, University of Energy and Natural Resources, PMB, Sunyani 3520, Ghana; nanaprempeh29@yahoo.com

**Keywords:** phosphorus fractions, river sediments, influencing factor, sequential extraction, Tiaoxi watershed, Hongze watershed

## Abstract

This study explored the spatial distribution of phosphorus fractions in river sediments and analyzed the relationship between different phosphorus fractions and their environmental influence on the sediments within different watersheds in Eastern China. River sediments from two inflow watersheds (Hongze and Tiaoxi) to Hongze and Taihu Lake in Eastern China were analyzed by the sequential extraction procedure. Five fractions of sedimentary phosphorus, including freely sorbed phosphorus (NH_4_Cl-P), redox-sensitive phosphorus (BD-P), bound phosphorus metal oxide (NaOH-P), bound phosphorus calcium (HCl-P), and residual phosphorus (Res-P) were all analyzed. The orders of rankings for the P fractions of the rivers Anhe and Suihe were HCl-P > NaOH-P > BD-P > NH_4_Cl-P and HCl-P > BD-P > NaOH-P > NH_4_Cl-P, respectively. For the rank order of the Hongze watershed, HCl-P was higher while the NH_4_Cl-P contents were significantly lower. The rank order for the Dongtiaoxi River was NaOH-P > HCl-P > BD-P > NH_4_Cl-P, and that of Xitiaoxi River was NaOH-P > BD-P > HCl-P > NH_4_Cl-P. Compared with the phosphorus forms of the Tiaoxi watershed, NaOH-P contents were significantly higher compared to HCl-P, which was significantly higher in the Hongze watershed. In comparison, NH_4_Cl-P contents were significantly lower in both. Variations may be attributed to differential discharge of the P form in the watershed due to land-use changes and urban river ambient conditions.

## 1. Introduction

Lake eutrophication is among the world’s most daunting environmental issues. Nutrient enrichment, in particular, phosphorus (P) and nitrogen (N), has been seen as a significant threat to the protection of coastal watersheds for over 30 years [1]. Phosphorus is very much a component of growth that restricts aquatic species and is the main driving factor for eutrophication in lake ecosystems [2,3]. Rapid population growth, industrialization, and increased agricultural production account for the massive release of untreated sewage and waste into the environment. Wastewater is created by most anthropogenic activities using water. When the overall demand for water rises, the quantity of wastewater generated and its overall pollution load continually rises around the world. The overwhelming bulk of wastewater is directly discharged into the environment without proper treatment, with adverse effects on human health, economic growth, natural biodiversity, and habitat quality [4,5]. Phosphorus (P) plays an important role in agricultural production as a dominant fertilizer input and as a source of surface water eutrophication [6]. It is a crucial component of plant life and can accelerate eutrophication (a decrease in dissolved oxygen in watercourses caused by a rise in mineral and organic nutrients) of rivers and lakes when in excess quantities [7]. As a consequence, the anthropogenic contribution of mobilized phosphorus (P) in watersheds flows into rivers and lakes, exacerbating the risk of surface water eutrophication [8].

Sediment is an essential source of nutrients and contributes nutrients to freshwater bodies. P in water has external and internal sources; P can come from external sources (agricultural and natural) or point sources (domestic effluents and industrial), while the internal source of P comes from the sediments within the water system [9]. Many small and large rivers in cities of Eastern China are polluted due to anthropogenic activities leading to several ecological issues in the aquatic environment.

Sequential extraction methods have been widely used in sediment phosphorous morphology studies in recent years. Sediment P is classified into various forms, such as exchangeable P, P bounded to calcium, P bounded to Al and Fe oxides, inorganic P (Inorg-P), and organic P (Org-p) [10,11,12,13,14]. The variations in the concentration of total P and its fractions in sediments is not uniform within the surface sediments (depths of 0–30 cm). This is due to the anthropogenic effects of phosphorus and the mechanisms of P release from sediment in the Hongfeng Reservoir [15].

Several authors have studied phosphorus fractions and their release in the sediments of rivers globally. The environmental influence of sediments as well as the characteristics of phosphorus, total phosphorus (TP), and dissolved total phosphorus (DTP) fractions of water and excess water in sediments in Xiangxi Bay were explored by Luo et al. [16]. At the same time, Song et al. [17] focused on the spatial distribution of P fractions in the Meiliang Bay sediment using the standard measurements and testing (SMT) sequential process. On the other hand, Zhang et al. [5] explored phosphorus properties of surface water in different river systems and their relationship with environmental impacts in Eastern China by SMT fractionation.

The sequential chemical extraction procedure was used by [18] to study the amounts and phosphorus forms in the Haihe River surface sediment. Phosphorus from anthropogenic activities in the watersheds gets into the rivers and lakes, which increases the risk of water eutrophication [8]. It is, therefore, important to identify P-sources (both externally and internally) to help control inputs of nutrients into freshwater systems.

While there is limited information about the characteristics of P and its environmental influence in the various watersheds, so far, no study has addressed the overall phosphorous content of the sediment and the concentrations of different phosphorous fractions as well as their influence factors in different watersheds. As such, Hongze and Tiaoxi watersheds were selected in this work because they have been significantly affected by anthropogenic activities such as agricultural, domestic, and industrial activities. The Hongze and Tiaoxi watersheds are of ecological importance to the Hongze and Taihu lakes, and many environmental protection initiatives such as domestic wastewater treatment and enforcement of China’s water pollution control law have made a great deal of effort to control pollution loads.

Studying the spatial variations and characteristics of P in the sediments in this study area will provide valuable information on the river sediments and their influence factors and can develop a theoretical basis for the management of the environment. The goal of this research was (1) to study the composition and spatial variation of phosphorus forms in sediment, (2) to evaluate the relationship between the P forms and the physicochemical properties, and (3) to study the environmental significance of P in the area of research.

## 2. Materials and Methods

### 2.1. Study Area

Situated in northwestern Jiangsu Province (33°06′–33°40′N, 118°10′–118°52′E), Hongze Lake is a relatively shallow lake with 1597 km^2^ of surface water area, maximum water depth of 4.37 m, mean water depth of 1.77 m, 27.9 × 10^8^ m^3^ of water storage capacity, and 354 km of coastline. With four distinct seasons, the lake region has monsoon climate characteristics, which is governed by the water body of the Hongze River. The mean annual rainfall is 925.5 mm, with the rainy season mostly between June and September [19,20]. With water transfer rates exceeding 11 times a year, the annual mean flow into the lake is 33 billion m^3^. Hongze Lake had been primarily collecting water supply in the top–middle sections of the Huaihe River urban wastewater and household sewage. In contrast, water contamination limited the function of the Hongze Lake environmental service. The lake covers six counties, namely Xuyi, Hongze, Sihong, Siyang, Huaiyin, and Jinhu. The Hongze watershed includes the Anhe River and Suihe River. They represent large variations in eutrophy and are anthropogenically influenced to varying degrees [21].

The Taihu Basin is situated in east China’s Changjiang Delta zone. In contrast, the Tiaoxi watershed is situated northwest of the province of Zhejiang with a latitude of 30°07′–30°41′N and a longitude of 119° 07′–119° 08′ E. The Tiaoxi watershed is part of the Taihu Basin and is an industrially developed region inside Zhejiang Province of China. The Tiaoxi watershed’s geomorphology from southwest to east and northeast ranges from mountains to hills, which are as high as 1500 m, and plain regions varying between and 3–5 m in height [21]. The length of the watershed is 157.4 km, and the catchment area measures more than 4570 km^2^. The Tiaoxi watershed (Dongtiaoxi and Xitiaoxi Rivers) is composed of two major tributaries, which converges at Bai Quetang Bridge in Huzhou city and then flows into the Taihu Lake. The annual runoff of Tiaoxi River is 14.93 × 10^9^ m^3^ and is one of the major tributaries of the Taihu Lake. The Hongze watershed includes the Anhe River and Suihe River. They display large variations in eutrophy and are anthropogenically influenced to varying degrees [21].

### 2.2. Sampling Sites

**Figure 1:**
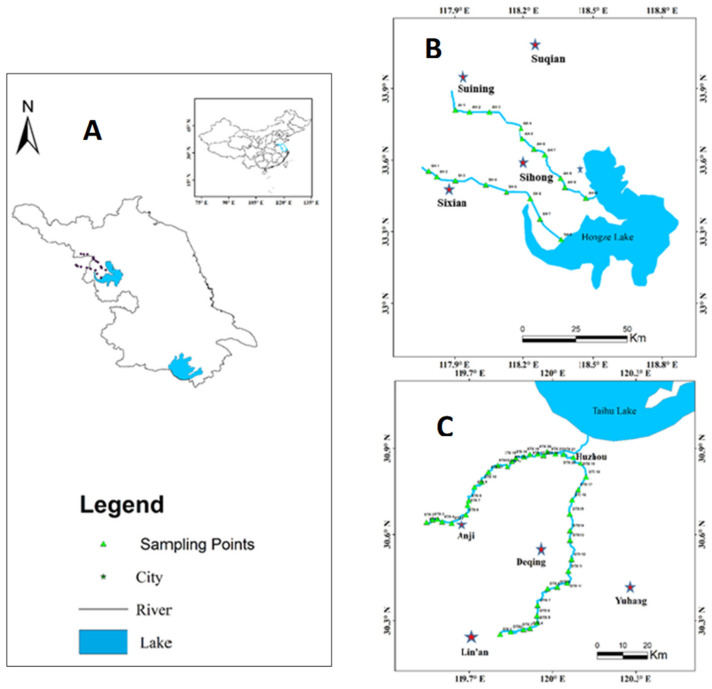
Map of the sampling sites showing (**A**) Northwest of Jiangsu province (**B**) Hongze watershed (**C**) Tiaoxi watershed.

A total of 60 river surface sediment samples (Figure 1) were taken from the sampling sites at the main sites and tributaries of the watersheds of Hongze and Tiaoxi. Sampling sites were distributed throughout the study area. Both the natural environment characteristics and spatial distribution of all forms of land use in the study area were considered. The grab sampler, obtained from Easy sensor institute, Nanjing China, was used to collect river sediment samples (depth = 0–10 cm) from upstream and downstream sites in the Tiaoxi and Hongze watersheds.

The collected sediment samples were promptly sealed in plastic bags made in polyethylene. All samples were placed in storage bottles and kept at a temperature of 4 °C. The samples were then taken to the laboratory for analysis. Samples were freeze-dried, grounded, and passed through a 20-mesh sieve for homogenization and analysis. From Figure 1, sample collection points in the Hongze watershed were labeled “AH1-AH10” and “SH1-SH8”, representing samples taken at Anhe River and Suihe River, respectively. The samples collected at the Tiaoxi watershed (DTX and XTX) were labeled “DTX1–DTX21” and “XTX1–XTX 21”, representing samples collected from Dongtiaoxi River and Xitiaoxi River, respectively.

The sediments were tested for total iron (Fe), aluminum (Al), magnesium (Mg), manganese (Mn), and calcium (Ca) using the ray fluorescence analyzer from Panaco (PW2440 type) purchased from Panaco Institute in the Netherlands. The organic matter (OM) was measured using the Walkey–Black technique in accordance with the work by Kim [22]. Total nitrogen (TN) was measured using the concentrated H_2_SO_4_ digestion technique [23]. Total phosphorus was measured using the ammonium molybdate spectrophotometry technique with the aid of the UV-1800 UV-Vis Spectrophotometer (Shimadzu Scientific Instrument, Columbia, MD, USA). Inorganic phosphorus (Pi) was analyzed using direct extraction with hydrochloric acid with a concentration of 1 M (time = 16 h) and tested using the molybdate blue method [24].

### 2.3. P Fractions in Sediment

**Figure 2:**
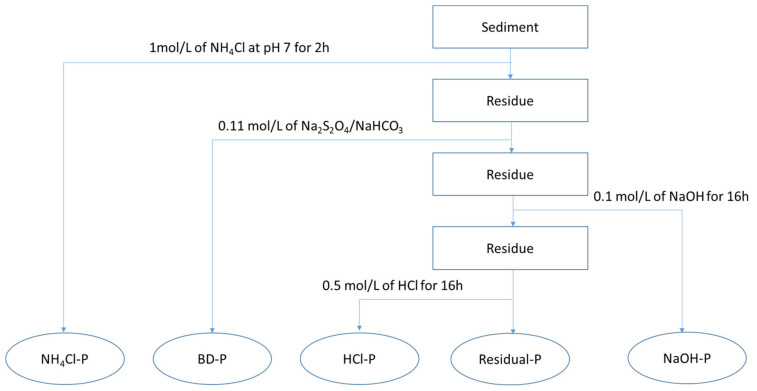
Sequential phosphorus (P) fractions scheme.

In this study, the inorganic P content fractions in the sediments were measured and analyzed. The different phosphorus contents were determined using the sequential extraction scheme, according to Psenner et al. [25] and Hupfer et al. [26]. The extraction process separated fractions of inorganic phosphorus (IP) in the sediment into freely sorbed P (NH_4_Cl-P), redox-sensitive P (BD-P), metal-oxide-bound (NaOH-P), and calcium-bound P (HCl-P) (Figure 2). The distinction between TP and IP is the remnant fraction P (Res-P), consisting of organic P and refractory P compounds.

### 2.4. Data Statistics and Analysis Methods

The principal component analysis (PCA) was carried out using the SPSS, AsiaAnalytics (Xi'an China)software kit, which is IBM Corp’s data processing platform. PCA can transform a significant number of possibly associated variables into a reduced set of independent variables called main components [21,27]. In this study, PCA was adopted to examine the factors that influence P-fraction variations in suspended and surface sediments in the Tiaoxi watershed. The detailed statistical analysis was performed using the statistical software program SPSS ver. 23.0, while illustrations were produced using the software Origin 9.0. OriginLab (Northampton, USA).

## 3. Results and Discussion

### 3.1. Characteristics of Phosphorus Fractions in Sediments

#### 3.1.1. Hongze Watershed

The chemical characteristics of Anhe and Suihe River sediments are presented in Table 1. Sediment properties such as Ca, Mn, Fe, OM, TN, and pH varied greatly in the two rivers. The contents of Ca, Mn, Fe, OM, and TN and the pH in Anhe River were 3.71–6.62%, 0.01–0.17%, 2.96–4.57%, 0.97–3.80%, 789.29–1310.21 mg kg^−1^, and 7.97–8.66, with averages of 5.75%, 0.06%, 4.18%, 1.85%, 1114.44 mg kg^−1^, and 8.18, respectively.

For Suihe River, the contents of Ca_,_ Mn, Fe, OM, and TN and the pH were 2.80–7.76%, 0.00–0.09%, 2.76–4.71%, 1.13–2.31%, 923.72–1398.44 mg kg^−1^, and 7.66–8.32, with averages of 5.03%, 0.05%, 3.83%, 1.68%, 118.34 mg kg^−1^, and 7.96, respectively.

In comparing the different watersheds, for Hongze watershed, the total nitrogen (TN) contents of Anhe and Suihe Rivers averaged 1114.44 mg kg^−1^ and 1182.34 mg kg^−1^, respectively. The range in concentration for TN content for Anhe and Suihe Rivers were 789.29−1310.21 mg kg^−1^ and 923.72–1398.44 mg kg^−1^, respectively. The findings showed that the concentration of TN in the Suihe River was greater than that of the Anhe River. In the case of total phosphorus (TP) contents, the Anhe and Suihe Rivers (both belonging to the Hongze watershed) averaged 841.52 mg kg^−1^, with a range of 514.71 mg kg^−1^ to 1078−86 mg kg^−1^, and 675.12 mg kg^−1^, with a range of 398.18 mg kg^−1^ to 1093.10 mg kg^−1^, respectively. The comparison results showed that the concentration of TP in Anhe River was higher than that of Suihe River.

Several studies have demonstrated that anthropogenic activities such as farming generate a lot of organic matter and may be responsible for the high total nitrogen (TN) and total phosphorus (TP) concentrations in a water system [28]. In this study, as indicated in Table 1, the high TP in Anhe River compared to Suihe River corresponded to the high OM content recorded in Anhe River compared to Suihe River. The average organic matter (OM) content in Anhe River increased by 10.1% compared to Suihe River, thus explaining the higher TP content recorded in Anhe River compared to Suihe River. This suggests that the physicochemical properties of the various sediments display different concentrations due to the differences in watershed characteristics and pollution sources.

#### 3.1.2. Tiaoxi Watershed

The chemical characteristics of Dongtiaoxi and Xitiaoxi River sediments are presented in Table 2. Sediment properties such as Al_2_O_3_, SiO_2_, OP, IP, TP, and TN varied greatly in the two rivers. The contents of Al_2_O_3,_ SiO_2_, OP, IP, TP, and TN in Dongtiaoxi River were 22.26–26.73%, 55.16–75.88%, 116.96–279.77 mg kg^−1^, 698.74–1059.86 mg kg^−1^, 815.70–1302.60 mg kg^−1^, and 1102.68–1658.69 mg kg^−1^, with averages of 24.18%, 65.85%, 199.56 mg kg^−1^, 856.87 mg kg^−1^, 1056.43 mg kg^−1^, and 1244.24 mg kg^−1^, respectively.

For Xitiaoxi River, the contents of Al_2_O_3_, SiO_2_, organic phosphorus (OP), IP, TP, and TN in the sediments were 20.90–27.01%, 65.13–76.92%, 221.45–385.00 mg kg^−1^, 888.96–1248.39 mg kg^−1^, 1133.64–1665.11mg kg^−1^, and 1186.03–1530.76mg kg^−1^, with averages of 24.09%, 71.10%, 308.16 mg kg^−1^, 1065.60 mg kg^−1^, 1373.76 mg kg^−1^, and 1438.00 mg kg^−1^, respectively. Thus, Xitiaoxi River is the most contaminated with relatively high IP, OP, TN, and TP contents.

In the Dongtiaoxi River, the contents of CaO, MnO_2_, and Fe_2_O_3_ in the sediments were 1.28–11.99%, 0.07–0.23%, and 5.95–7.60%, with averages of 5.09%, 0.17%, and 6.89%, respectively. For Xitiaoxi River, the contents of CaO, MnO_2_, and Fe_2_O_3_ in the sediments were 0.66–6.05%, 0.06–0.23%, and 4.92–7.76%, with averages of 1.58%, 0.11%, and 6.52%, respectively. These results indicate a higher concentration in the Dongtiaoxi River sediments compared to that of Xitiaoxi sediments, attributed to discharge from urban effluent, which affects the chemical behavior of phosphorus in the fluvial system.

There are some significant variations in chemical properties between the rivers Dongtiaoxi and Xitiaoxi. These differences were expected since the Dongtiaoxi River has higher pollutant inputs than the Xitiaoxi River (Table 2). These findings suggest industrial effluent discharges of some of the major components of river sediment that influence the chemical activity of phosphorus in the river system and, thus, the profound variation in the Xitiaoxi River.

### 3.2. Distributions and Spatial Variations of Different P Forms in River Sediments

**Figure 3:**
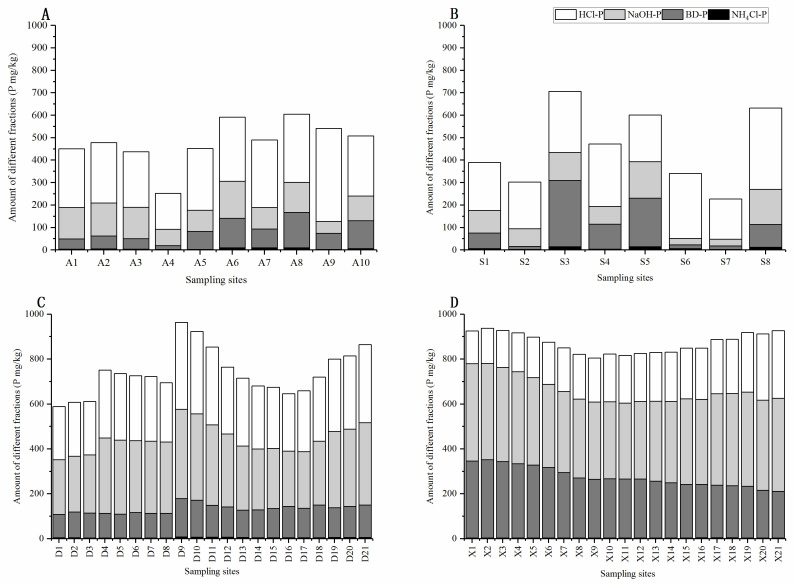
The concentration of different P fractions in different sediments of rivers: (**A**) Anhe, (**B**) Suihe, (**C**) Dongtiaoxi, and (**D**) Xitiaoxi.

The sediment phosphorus content was controlled by factors such as land use. The phosphorus content showed variations in land usage (anthropogenic activities) [29]. The average relative distribution of different P fractions in the sediment of different watersheds is presented in Figure 2 and Figure 3. The contents of TP and different P fractions studied significantly varied.

The P fractions considered were inorganic P forms, including NH_4_Cl-P, BD-P, NaOH-P, and HCl-P as well as organic P fractions. The spatial distribution of the fractions varied considerably in different reaches of the different watersheds, with a general increasing trend along with the river inflow.

The average contents of NH_4_Cl-P, BD-P, NaOH-P and HCl-P of Dongtiaoxi River were 4.93 mg kg^−1^, 128.55 mg kg^−1^, 309.52 mg kg^−1^, and 295.96 mg kg^−1^, respectively, while Xitiaoxi River recorded 3.40 mg kg^−1^, 271.72 mg kg^−1^, 384.10 mg kg^−1^, and 213.09 mg kg^−1^, respectively (Figure 3C,D).

The various P fractions in sediments were significantly influenced by land-use changes and the geological layout of the watercourse [30]. For the Hongze watershed, the average contents of NH_4_Cl-P, BD-P, NaOH-P, and HCl-P in the Anhe River were 5.65 mg kg^−1^, 81.53 mg kg^−1^, 131.50 mg kg^−1^, and 278.47 mg kg^−1^, respectively (Figure 3A). The sequence of P fractions was NH_4_Cl-P > BD-P > NaOH-P > HCl-P. The averages of Suihe River were 7.82 mg kg^−1^, 104.86 mg kg^−1^, 95.07 mg kg^−1^, and 251.32 mg kg^−1^ respectively (Figure 3B). The common sources of phosphorus in the river sediments follow the following order: NaOH-P > HCl-P > BD-P > NH4Cl-P for Dongtiaoxi River and NaOH-P > BD-P > HCl-P > NH4Cl-P for Xitiaoxi River.

The ranking order of Anhe River P fractions is HCl-P > NaOH-P > BD-P > NH_4_Cl-P, whereas Suihe River’s is HCl-P > BD-P > NaOH-P > NH_4_Cl-P. These variations are due to various variables, such as particle size composition, redox potential, acidity, as well as environmental conditions associated with soil and sediment [31,32,33,34]. Sediment phosphorous content was high because of its high binding potential with sediment and minerals such as calcium, aluminum, and iron [35].

NH_4_Cl-P refers to loosely sorbed P in sediments. This fraction can contain dissolved P in pore water [27]. NH_4_Cl-P is a seasonal variable phosphorous dissolved in interstitial water [31]. In Hongze watershed, the Anhe River concentrations of NH_4_Cl-P in the sediments ranged from 2.55 mg kg^−1^–9.77 mg kg^−1^, with an average of 5.65 mg kg^−1^. In contrast, Suihe concentrations were 2.37 mg kg^−1^–14.78 mg kg^−1^, with an average of 7.82 mg kg^−1^ for all sediment samples.

The highest concentration of NH_4_Cl-P in the sediment of the Suihe River is the maximum concentration of sedimentary inorganic P. This fraction of P makes up for 1.56% of the sedimentary inorganic P in the Hongze watershed. BD-P is a redox-sensitive P fraction, consisting mainly of Mn compounds and P bound to Fe hydroxides [36].

The BD-P reagent reduces the oxidized species of iron and manganese, thereby releasing the phosphorous adsorbed oxide of the two metals. Iron-bound P, determined by BD extraction, has been proven to provide the best estimate of internal P loading. BD-P represents the redox-sensitive P forms that are considered a potential mobile pool of P and are algal available [31].

NaOH-P is exchangeable, including P bound to metal oxides, mainly of Al and Fe [27]. NaOH extractable phosphorus may be discharged at the sediment-water interface for the growth of phytoplankton under an anaerobic environment [37]. Active Fe and Al were considered the key adsorbents of P in sediments [38]. BD-P and NaOH-P are principally bound to Fe and Al, notably in their amorphous and active forms.

HCl-P is a low pH-sensitive P fraction and is presumed to consist entirely of apatite P, including P bound to carbonates and traces of organic hydrolyzable P [27]. Calcium bound P is a reasonably stable sedimentary fraction of P and leads to a lasting burial P in sediments [27]. This P fraction was considered a relatively stable IP fraction in sediment [27,39,40].

### 3.3. Bioavailable Phosphorus (BAP) in the Sediments

**Figure 4:**
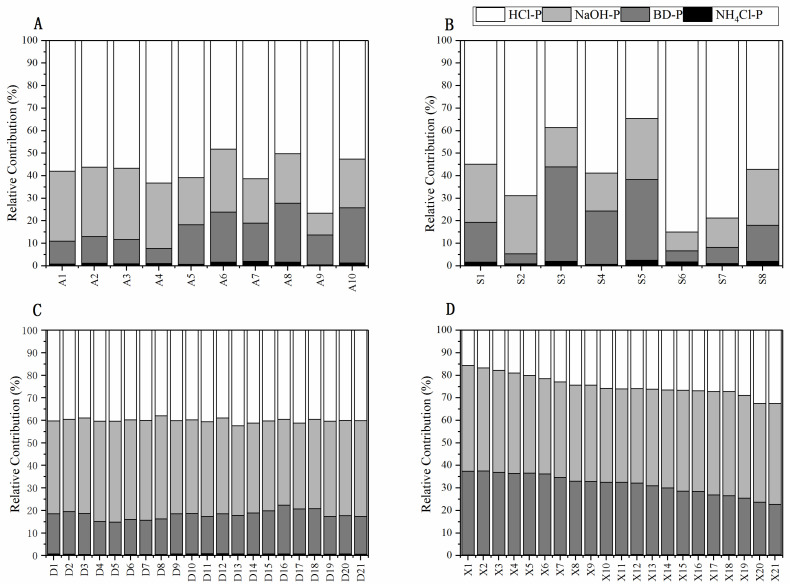
The relative contribution of different P fractions to inorganic phosphorus (IP) in different sediments of rivers: (**A**) Anhe, (**B**) Suihe, (**C**) Dongtiaoxi, and (**D**) Xitiaoxi.

Bioavailable phosphorus can be converted to be usable by physical, chemical, and biological cycles, depending on the chemical environment. Environmental factors such as pH and redox potential influence mobilization. Bioavailable phosphorus is calculated as the sum of available P and P, which can be transformed by natural phenomena into free form. The order of Bioavailable Phosphorus (BAP) content on the four banks of the river from the comparison of phosphorus content in different parts is Dongtiaoxi (269.91 mg kg^−1^) > Suihe (233.30 mg kg^−1^) > Anhe (225.53 mg kg^−1^) > Xitiaoxi (167.88 mg kg^−1^). The associated sediment order is Xitiaoxi (311.61 mg kg^−1^) > Suihe (280.43 mg kg^−1^) > Dongtiaoxi (251.00 mg kg^−1^) > Anhe (224.72 mg kg^−1^). The ratios of BAP to IP follow the order from the comparison of phosphorus types in various river sections: Dongtiaoxi (68.03%) > Suihe (69.60%) > Anhe (66.05%) > Xitiaoxi (63.62%). As for sediments, we have Xitiaoxi (75.78%) > Suihe (71.59%) > Dongtiaoxi (74.21%) > Anhe (66.96%) (Figure 4). Compared to previous studies, Lane and Autrey [41] indicated that forest wetlands generated greater organic matter content than evolving wetlands ecosystems, which was also associated with higher phosphorus sorption [42]. Reported urban districts with higher population density and relatively low nutrient surplus and those cities or industrial regions with low population growth and high nutrient excess in the soil surface are situated in the Fujian province of China. However, our study findings revealed that sparse population and broad forestry coverage additionally inhibit a lot of terrestrial BAP content as well as the Dongtiaoxi catchment’s phosphorus production, which ultimately preserves quality of water.

In the current study, the scheme proposed is BAP = NH_4_Cl-P+ NaOH-P + BD-P. The potentially bioavailable phosphorus can contribute significantly to local primary production when this fraction enters the water column. As such, assessment of the water ecological ecosystem should recognize not only the phosphorus content but also the phosphorus forms.

### 3.4. Correlation Analysis of Phosphorus in River Sediments Composition of the Different Watersheds

The connection between the different P forms in the sediments and their physicochemical properties was evaluated to understand the sediment’s effect and composition on the distribution of P forms in the sediment (Table 3 and Table 4). Table 3 demonstrates the relationships between phosphorus fractions and sediment characteristics in Hongze watershed. Al in the Anhe River positively (*p* < 0.01) correlated with NH_4_Cl-P, BD-P, and HCl-P. Ca positively (*p* < 0.01) correlated with BD-P, and Fe significantly correlated with NaOH-P positively. HCl-P significantly (*p* < 0.05) correlated with Al, Ca, and pH positively. In the Suihe River, Al, Fe, OM, and TP were highly and positively connected with NH_4_Cl-P, BD-P, and NaOH-P. Ca and pH were significantly (*p* < 0.05) correlated with HCl-P positively. This demonstrates that BD-P and NH_4_Cl-P may be released easily from the sediment in Suihe River, and they were the principal fractions of the released phosphorus sources in the river sediments.

Also, the correlation between the different fractions of P in the sediment and the physicochemical characteristics of the sediments are presented in Table 4. The correlation result reveals that IP and TP have a strongly significant (*p* < 0.01) correlation with NaOH-P and HCl-P in sediments. In contrast, the total organic carbon (TOC) and MnO_2_ in Dongtiaoxi River had a significant negative (*p* < −0.01) connection to NH_4_Cl-P and BD-P.

TN had a significant (*p* < 0.01) positive correlation with NH_4_Cl-P, BD-P, NaOH-P, and HCl-P. There was no significant relationship between Al_2_O_3_, SiO_2_, Fe_2_O_3_, and OP. Meanwhile, in the Xitiaoxi River, BD-P negatively (*p* < −0.01) correlated with CaO and TN. HCl-P contents showed a significantly positive (*p* < 0.05) correlation with CaO. A higher TOC concentration in the sediment, coupled with smaller particle size, contributes to a more robust capacity of P adsorption [13].

### 3.5. Environmental Significance of Phosphorus Distribution and Implications for Watershed Management

The findings obtained above demonstrate that the distribution of phosphorous forms in river sediments has intrinsic connections. Human activities meanwhile threaten the distribution of TP and phosphorus fractions in riparian soils and river sediments, thus having implications for the management of the different watersheds flowing into the lake. Landscape patterns have been characterized by certain geographical factors, such as topography, climate, geology, and land use or types of land cover [43,44]. Rivers are especially susceptible because of their nearness to cities and towns and sensitivity to land-use changes [45,46].

Watersheds appeal to many environmental managers as they have well-defined boundaries that allow for the determination of relative water and solute budgets. Many of the watersheds are altered by humans, atmospheric deposition, forestry and floodplain management, eutrophication, and other types of biogeochemical hydrological changes in freshwater [47]. These transformations can either reduce or increase their performance under increasing environmental conditions.

Differences in water and sediment in watershed lakes may lead to the development of substantially different lake ecosystems. The watershed variables were found to be the consolidated factors in the upstream landscape of the sampling stations and the geomorphological conditions of the sampling sites as well as the mean slope and distance from the river channel [48,49]. The challenge is to be able to focus on much research for a broader understanding of the watershed, which is useful to environmental managers [49].

The behavior of anthropogenic P in a watershed given significant human activity and landscape topography, inputs of P, and exports across the river basin are influenced along such gradient [50]. Nutrient concentration in rivers is of vital importance to the ecosystems of the river itself. At the same time, the transfer of nutrients by the river is indeed also important for any other receiving media [51].

Rising human-induced operation coupled with current land use will enhance pollutant loads, such as nutrients and microbes, into water sources, which can threaten human health [52] and rainfall events [53]. This may further increase the loading of contaminants due to the emergence of runoff from both agricultural and residential areas as a result of livelihood activities, including the use of compost as fertilizer and animal grazing at river banks [54]. If environmental managers determine that P is a problem in the receiving watershed, they can manage to maintain more exceptional aerobic conditions, thereby reducing the release of P. Additionally, a significant release of P from different watersheds under aerobic conditions has implications for water quality. Freshwater discharges have been impacted by water withdrawals for urbanization, irrigation, and aquaculture, reducing the dilution potential of the estuary and increasing the harmful impact of nitrogen emissions from anthropogenic sources [55]. This confirms that intensive anthropogenic practices will raise phosphorus biodisponibility.

In comparison, the forest coverage rate is tremendous in the entire basin (approximately 75%) [55]. Urbanization has damaged the soil’s nutrient equilibrium [42] and started to trap nutrient materials in lake sediments. To a certain degree, the Tiaoxi increasing nutrients would degrade the quality of the water in the Taihu downstream reaches. Therefore, greater emphasis should be allocated to the regulation of land use and waste disposal in the basin and appropriate consideration should be allocated to water quality.

## 4. Conclusions

This study highlighted the characteristics of P fractions in river sediments and its influence factors in Hongze and Tiaoxi watersheds. The spatial distribution characteristics of the TP and phosphorus fractions in the sediments differed significantly in the Tiaoxi watershed. NH_4_Cl-P and BD-P can be easily released from sediments and can contribute mainly to the release of phosphorus in the sediment of the Hongze watershed. The phosphorus fraction of CaO, MnO_2_, and Fe_2_O_3_ in Dongtiaoxi River was higher than in the Xitiaoxi River, whereas SiO_2_, TP, OP, IP, and TN in Xitiaoxi River were mainly phosphorus pollution. Influence factor P in sediments was controlled by chemical action. The average forms of phosphorus in river sediments follow the order: NaOH-P > HCl-P > BD-P > NHCl_4_ -P. The potential of P adsorption was higher, and the sediment had a high adsorption rate for NaOH-P and HCl-P in different watersheds. This is associated with the acid soil of the Hongze Basin granite region. However, the distributions of phosphorus sources in both watersheds are not necessarily consistent between river sediments.

Human impacts alter the distribution of phosphorus types in floodplain soils (BD-P, NaOH-P, and HCl-P) and hence influence their distribution in river sediments. Rapid urbanization and nutrient elements are beginning to aggregate in river sediments, and appropriate consideration should be allocated to water quality monitoring in Hongze Basin. Furthermore, there is a need to improve management of watersheds such as the Xitiaoxi River if the watershed as a whole is to be adequately controlled.

## Figures and Tables

**Table 1:** Physical and chemical characteristics of sediment properties of Anhe River and Suihe River.

Contents	Anhe River Sediment Mean (Range)	Suihe River Sediment Mean (Range)
Al (%)	10.11 (7.52–12.07)	10.20 (7.92–14.05)
Ca (%)	5.75 (3.71–6.62)	5.03 (2.80–7.76)
Mn (%)	0.06 (0.01–0.17)	0.05 (0.00–0.09)
Fe (%)	4.18 (2.96–4.57)	3.83 (2.76–4.71)
OM (%)	1.85 (0.97–3.80)	1.68 (1.13–2.31)
TN (mg kg^-1^)	1114.44 (789.29–1310.21)	1182.34 (923.72–1398.44)
TP (mg kg^-1^)	841.52 (514.71–1078.86)	675.12 (398.18–1093.10)
pH	8.18 (7.97–8.66)	7.96 (7.66–8.32)

OM, organic matter; TN, total nitrogen; TP, total phosphorus.

**Table 2:** Physical and chemical characteristics of sediment properties of Dongtiaoxi River and Xitiaoxi River.

Contents	Dongtiaoxi River Sediment Mean (Range)	Xitiaoxi River Sediment Mean (Range)
TOC(%)	2.25 (1.76–2.82)	2.25 (0.68–3.85)
OM (%)	1.95 (0.6–2.1)	2.31 (0.7–3.1)
Al_2_O_3_%	24.18 (22.26–26.73)	24.09 (20.90–27.01)
SiO_2_%	65.85 (55.16–75.88)	71.10 (65.13–76.92)
CaO%	5.09 (1.28–11.99)	1.58 (0.66–6.05)
MnO_2_%	0.17 (0.07–0.23)	0.11 (0.06–0.23)
Fe_2_O_3_%	6.89 (5.95–7.60)	6.52 (4.92–7.76)
TP (mg kg^−1^)	1056.43 (815.70–1302.60)	1373.76 (1186.03–1530.76)
OP (mg kg^−1^)	199.56 (116.96–279.77)	308.16 (221.45–385.00)
TN (mg kg^−1^)	1244.24 (1102.68–1658.69)	1438.00 (1186.03–1530.76)
IP (mg kg^−1^)	879.3 (698.74–1059.86)	1068.67 (888.96–1248.39)

OM, organic matter; TOC, total organic content; TN, total nitrogen; TP, total phosphorus; OP, organic phosphorous; IP, inorganic phosphorous.

**Table 3:** Pearson’s correlation coefficient of phosphorus fractions of sediments in Hongze watershed.

Contents	Anhe River				Suihe River			
	NH_4_Cl-P	BD-P	NaOH-P	HCl-P	NH_4_Cl-P	BD-P	NaOH-P	HCl-P
Al	0.633 *	0.854 **	0.151	0.649 *	0.813 *	0.976 **	0.708 *	0.332
Ca	0.484	0.662 *	0.041	0.787 **	0.267	0.226	0.086	0.924 **
Mn	0.137	0.202	−0.213	−0.303	−0.403	−0.496	−0.318	0.679
Fe	0.582	0.421	0.939 **	−0.153	0.794 *	0.770 *	0.976 **	0.261
OM	0.200	0.085	0.412	0.191	0.931 **	0.907 **	0.817 *	0.285
TN	0.395	0.469	0.555	0.496	0.408	0.141	0.351	0.506
TP	0.506	0.470	0.523	0.401	0.932 **	0.867 **	0.907 **	0.321
pH	0.211	0.469	−0.366	0.878 **	0.133	−0.063	0.004	0.935 **

*p* < 0.05 * and *p* < 0.01 **, * correlation is significant at 0.05 and ** correlation is significant at 0.01.

**Table 4:** Pearson’s correlation coefficient of phosphorus fractions of sediments in Tiaoxi watershed.

Contents	Dongtiaoxi River				Xitiaoxi River			
	NH_4_Cl-P	BD-P	NaOH-P	HCl-P	NH_4_Cl-P	BD-P	NaOH-P	HCl-P
TOC	−0.808 **	−0.770 **	−0.087	−0.405	0.168	−0.198	−0.031	0.279
Al_2_O_3_	−0.342	−0.327	0.003	−0.095	−0.015	0.267	0.072	−0.140
SiO_2_	−0.033	−0.116	0.281	0.237	−0.038	0.098	−0.029	−0.290
CaO	0.077	0.225	−0.202	−0.184	−0.037	−0.449 *	0.251	0.680 **
MnO_2_	−0.539 *	−0.461 *	−0.240	−0.373	0.062	0.004	−0.085	0.080
Fe_2_O_3_	−0.330	−0.257	−0.017	−0.075	0.409	−0.153	−0.157	0.152
IP	0.505 *	0.346	0.785 **	0.667 **	0.372	−0.232	0.181	0.154
OP	−0.369	−0.397	0.327	0.019	−0.235	0.059	−0.047	0.004
TP	0.245	0.113	0.726 **	0.524 *	0.327	−0.257	0.200	0.197
TN	0.719 **	0.754 **	0.843 **	0.862 **	0.399	−0.502 *	−0.073	0.421

*p* < 0.05 * and *p* < 0.01 **, * correlation is significant at 0.05 and ** correlation is significant at 0.01.
